# Diameters of the Fœtal Head, from Measurements Made in the Dublin Lying-in Hospital

**Published:** 1851-10

**Authors:** Addinell Hewson

**Affiliations:** Pennsylvania Hospital; Philadelphia


*Diameters of the Fœtal Head, from measurements made in the Dublin Lying-in Hospital.* By ADDINELL HEWSON, M. D., of Philadelphia. Communicated in a letter to Dr. MEIGS.

DEAR SIR :—Knowing the great interest which you always take in everything connected with medical science, and particularly with the branch of Obstetrics, I have availed myself of a short relaxation from my hospital duties, to draw up for you the results of some measurements of fœtal crania, which I made last Spring whilst an interne of the Dublin Lying-in Hospital. It affords me particular pleasure to communicate the results of these observations to you, as it was from the perusal of your recent work on Obstetrics that I was induced to make them.

On comparing the estimates of the diameter of the fœtal head which you have given, with those contained in foreign works on Obstetrics, a great difference will be observed. Your diameters are far greater than those given by any foreign author. The question therefore presented itself to my mind, is the estimate given by those abroad too low, or is there really an ethnological difference? The accuracy and extent of your own observations precluded all thought of your estimates not being correct, and I therefore availed myself of the occasion which presented, to ascertain, as well as I could, wherein consisted the difference.

For this purpose I extended my observations to one hundred and sixty-six children, born in the hospital, between the 10th of March and the 13th of April last. I did not select cases, but made my measurements promiscuously, independent of age or sex, of every child born alive, and at full term, in the Institution, between those dates.

I employed in making these measurements, a pair of delicate turner's calipers, admitting of accurate adjustment, and an ivory scale marked to the twentieth and fiftieth of an inch. Each measurement was made with the greatest care, within twenty-four hours after the birth of the child, and registered at the time.

The sum of my measurements of the biparietal diameter, was six hundred and three inches and eighty-eight hundredths, which gives a mean of three inches and six tenths, for that diameter. The sum of the occipito-frontal diameters was seven hundred and seventy-seven inches, and seventy-seven hundredths; the mean, four inches and sixty-eight hundredths. The sum of the occipito-mental diameters was eight hundred and eighty-seven inches and eighty-three hundredths; the mean, five inches and twenty-eight hundredths.

That you may consider these averages very just, I will mention the fact that there was not a very great range between the measurements. Thus, for instance, in the 166 biparietal diameters, but one exceeded four inches, (the mean being 3 6-10th inches.) You mention having met with sixty-eight exceeding that number in a series of one hundred and fifty measurements, the mean of which was three inches and eleven twelfths. The occipito-frontal diameter was five inches in six, out of my hundred and sixty-six, and in fifteen it exceeded that—the greatest being five and two-tenths. The occipito-mental attained six inches in three cases out of the hundred and sixty-six, in one other it reached six inches and one-tenth.

Having thus detailed to you the results of my measurements, permit me to draw your attention to the manner in which they will compare with the estimates given in standard foreign works. The followiug table contains the estimates given by some of the best English and French authorities. I have reduced the fractions in their figures to decimals, as my own are given in that scale. To the end of the table I have added my own estimates, that you may see at a glance how they compare:

| | *Bi-parietal.* | *Occipito-frontal.* | *Occipito-mental.* |
|---|---|---|---|
| Baudeloque, . . . | 3.34 to 3.50 . . | 4.50 . . | 5.50 |
| Velpeau, . . . . | 3.50 . . | 4 about . . | 5 |
| Cazeaux, . . . . | 3.50 to 3.75 . . | 4.25 to 4.50 . . | 5.25 |
| Burton, . . . . | 3.50 . . | 4.30 . . | 5.60 |
| Ashwell, . . . . | 3.50 . . | 4.50 . . | 5.25 |
| Murphy, . . . . | 3.50 . . | 4.50 . . | 5 |
| Churchill, . . . . | 3.50 to 4 . . | 4 to 4.50 . . | 5 |
| My own, . . . . | 3.60 . . | 4.68 . . | 5.25 |

You can readily see that, although mine are the highest, there is not a great difference amongst them all, but, that this difference is greater when you compare my estimate with those of Baudeloque and Velpeau. M. Cazeaux has not, in reality, given a mean, but has only given the points between which it may be found, and I might with propriety have omitted his measurements altogether, had I not wished to give you an opportunity of comparing mine with those of the most recent of French authorities. I have also very much to regret that Dr. Churchill has done the same, for he is the best of authorities in Ireland, and his well known accuracy of observation would, I believe, by its weight, have confirmed my results.

Now let me compare my results with those of your own observations in this country, and that such a comparison may be a perfectly just one, I will take the mean of a series of my measurements, equal in number to that of which you have given the mean.

You give the mean of a hundred and fifty measurements for the biparietal diameter, as three inches and eleven-twelfths, or 3 inches 88-100ths. The mean of the first hundred and fifty of my measurements, is 3 inches 63-100ths, the differerence is 25-100ths, or precisely a quarter of an inch. Your mean for the occipito-frontal diameter, from the same number of measurements, is four inches and ten lines, or four inches 83-100ths. My mean is 4 inches and 68100ths; the difference is 15-100ths, or about 1-7th of an inch.

You give the mean occipito-mental diameter, from 126 measurements, as five inches and five-tenths. The mean of the first 126 of my measurements of that diameter, is five inches and 36 hundredths; the difference, 14-100ths, of an inch, nearly the same as for the occipito-frontal diameter.

Thus you see that there is a very essential difference between our results, a difference too great to be attributable to inaccuracy on my part alone. I am conscious of having taken the greatest care in all my observations, and although my estimates are higher than those to be found in any foreign work, still they lead to the conclusion that there is really some other cause than inaccuracy to account for the difference; and may we not seek for it in an ethnological difference in the cranial development of the fœtus? This is certainly a question of interest, but it is not one which a single series of observations like my own can solve. With much respect I remain,

Yours sincerely,

ADDINELL HEWSON.

*Pennsylvania Hospital, Sept.* 13*th*, 1851.

